# To know the objective is not (necessarily) to know the objective function

**DOI:** 10.1186/s13040-018-0182-8

**Published:** 2018-10-04

**Authors:** Moshe Sipper, Ryan J. Urbanowicz, Jason H. Moore

**Affiliations:** 1 Institute for Biomedical Informatics, University of Pennsylvania, Philadelphia, 19104-6021 PA USA; 2 Department of Computer Science, Ben-Gurion University, Beer Sheva, 8410501 Israel

## Editorial

Finding the *objective* (i.e., goal or global optimum) in machine learning (ML) and related domains, such as evolutionary algorithms (EAs), invariably involves the definition of an *objective function*, which is the function we want to minimize or maximize [1]. Any objective function implicitly defines an optimization landscape, which is often deceptive, admitting many local optima. “The problem,” wrote Lehman and Stanley [2] a decade ago, “is that the objective function does not necessarily reward the stepping stones in the search space that ultimately lead to the objective.”

**Fig. 1:**
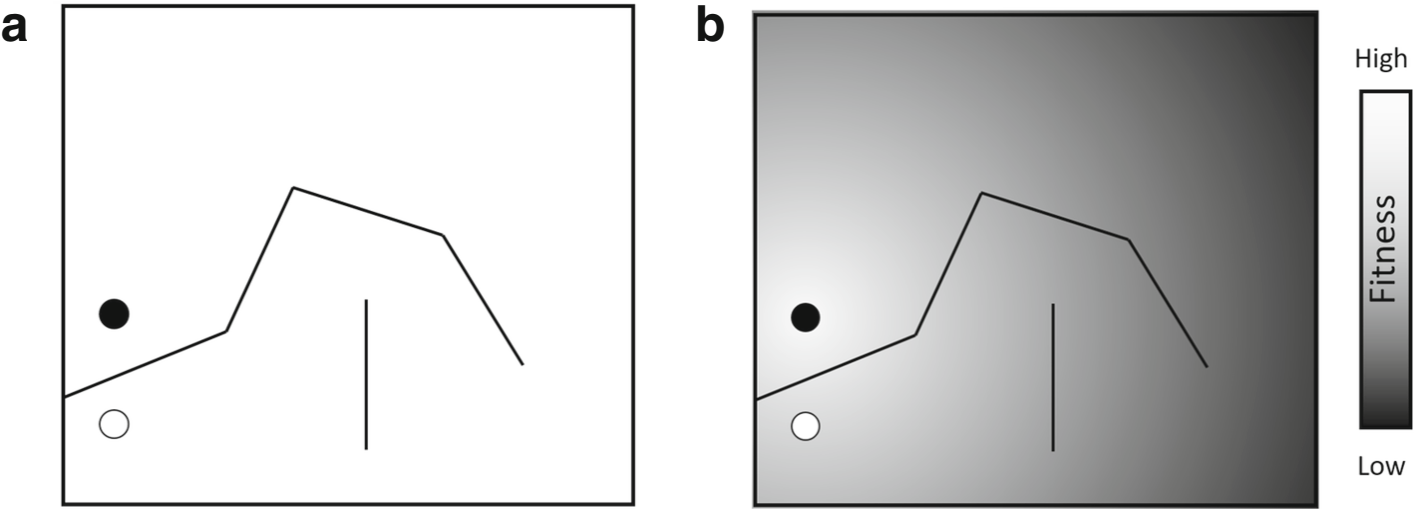
**a** Simple maze (based on [2]), wherein a robot begins at the empty circle and must make its way to the full circle (objective). **b** The maze’s fitness landscape, which emerges from a simple objective function: the inverse linear distance from the objective

Consider the maze of Fig. 1a, wherein the challenge is to evolve a robotic controller (i.e., a model that determines movement) such that the robot, when placed in the start position, is able to make its way to the goal (the controller’s representation is not essential to our argument).

It seems intuitive that the fitness *f* of a given robotic controller be defined as a function of the distance from the robot to the objective at the end of an evaluation, as done by [2] (see Fig. 1b). However, reaching the objective may be difficult since the robot starts with a relatively high fitness (because it is already close to the goal) and it will likely find itself stuck in a local optimum. Reaching the global optimum implies the acceptance of reduced fitness over the course of searching for an optimal controller. This is at odds with a standard search algorithm (be it ML or EA), which in practice is driven to optimize the objective function rather than find the best way to reach the objective. Another way to frame this problem is that while an optimal solution may be representable by the controller, it may not be learnable given this simple objective function [3]. For EAs, learnability translates to *evolvability* [4].

A similar example of this problem—*conflating* the objective with the objective function—from the computational genetics domain can be found in the application of machine learning to the modeling of genetic variables for the prediction of phenotypic outcome. The identification of underlying epistasis (i.e., gene-gene interactions) among genetic variables that represent a global optimum might be similarly confounded by an objective function that assumes that simpler univariate effects can serve as building-block stepping stones for identifying a complex multivariate association. Another perspective on this problem in this same domain is that while the chosen objective function may lead to modeling the most predictive variables, these are often likely to be variables that are simply associated with disease as local optima, while the underlying causal variants are being missed as the true global optimum of the search. In this scenario, the practitioner would not have the prior knowledge needed to define the best objective function to reach the global optimum. Yet another scenario where this might occur is in noisy problems where the maximally achievable prediction accuracy is not known a priori. Since ML algorithms are almost always driven to optimize model training accuracy, they are bound to overfit the data (at least to some degree) in noisy problems. In situations like this, the global optimum may be a model with a lower accuracy.

The conflation problem is distinct from the topic of dynamic fitness, wherein the problem itself (along with the global optimum and/or the fitness landscape) changes during run-time [5]. The prediction of weather patterns is in line with this challenge. Further, multi-objective optimization, where there is more than one objective to optimize at a time, is also distinct [6]. However, both of these challenges are vulnerable to this conflation of objective and objective function.

Lehman and Stanley [2] responded to this objective-function conundrum through what they called novelty search, which *ignores* the objective and searches for behavioral novelty (using a novelty metric that requires careful consideration). However, fundamental novelty search lacks an underlying incentive to identify the global optimum efficiently, or at all. Thus, while it can be an effective approach to avoiding local optima and discovering new candidate solutions, it does not solve the conflation problem discussed herein.

Perhaps the problem lies with our ignorance of the *right* objective function. To that end, Urbanowicz et al. [7] proposed an adaptive fitness landscape for genetic algorithms that updated the underlying objective function over the course of model training. While this was successful in reducing overfitting, this approach was still limited by the fact that a single value of fitness, determined by the objective function, was tasked both with identifying the optimal solution, as well as finding the building blocks to reach that optimal solution.

We propose a different strategy to overcome the conflation problem: Rather than seek out novel behavior or rely on a single fitness metric (evolvable or not) to evaluate both the “journey” and the “destination”, might it be more effective to separate the optimization of the solution from the optimization of the (fitness) function that will lead to a global optimum? Returning to the robot example, just because the robot’s objective is to reach the goal does not necessarily imply that the fitness function is distance-to-goal. Perchance evolvability deserves its own objective function? This putative function might, e.g., combine the distance to the goal, the distance from the start position, the distances to the various walls, the number of times the robot hits walls, and so forth. Thus we are now left with the task of finding this better objective function, either manually or—perhaps more intriguingly—through some automated means; after all, if we are searching for a good objective function, why not employ a search algorithm?

We conclude with a final speculation that co-evolution [8] might offer the key to simultaneously explore solution optimality and objective-function optimality.

## Abbreviations

EA: Evolutionary algorithm
ML: Machine learning
